# The Prevalence and Associated Factors of Hepatitis B and C Virus in Hemodialysis Patients in Ibb Governorate, Yemen

**DOI:** 10.7759/cureus.70112

**Published:** 2024-09-24

**Authors:** Radwan H. Ahmed, Nada Al-Nagar, Ibrahim Al-Subol, Rehab Al-Wahbi, Manar Al-Sabahi, Mohamed Al-Sabahi, Khaled Al-Sabahi, Asmaa Shomasi, Abdulmalik Al-Hamodi

**Affiliations:** 1 Department of Medical Laboratory, Faculty of Medicine and Health Sciences, University of Science and Technology, Sana'a, YEM; 2 Nephrology Unit, Al-Thawra General Hospital, Ibb, YEM; 3 Department of Medicine, Faculty of Medicine and Health Sciences, University of Science and Technology, Sana'a, YEM

**Keywords:** hbv, hcv, hemodialysis, viral infection, yemen

## Abstract

Introduction

Hepatitis C virus (HCV) and hepatitis B virus (HBV) among patients receiving hemodialysis (HD) remain a major public health problem. However, information is limited about these infections among HD patients in Yemen. This study aimed to estimate the prevalence and associated risk factors of HBV and HCV infections among HD patients in the Ibb governorate, Yemen, and to identify the risk factors for infections in such patients.

Methods

A cross-sectional study of 374 patients with renal failure who regularly underwent HD at the Al-Thawra Hospital in Ibb city, Yemen, was performed after they agreed to participate and signed informed consent. Enzyme-linked immunoassay (ELISA) was used to test the serum levels of anti-HCV antibodies and hepatitis B surface antigen (HBsAg). Patient data (demographic characteristics and risk factors) were collected via an interview questionnaire and medical records. Logistic regression analysis and chi-square tests were used to analyze the results.

Results

The overall prevalence of HCV was 31% (n=116), whereas that of HBV was 6.15% (n=23). Three (0.8%) patients had both HCV and HBV. The logistic regression analysis revealed a significant association between an increased number of units of blood transfused (OR = 3.1; 95% CI: 1.7-5.6; p < 0.001), a long duration of dialysis (OR = 3.2; 95% CI: 1.9-5.2; p = 0.001), and HCV infection in HD patients. On the other hand, a history of cupping therapy (Hijama) was significantly associated (OR = 3.4; 95% CI: 1.3-8.77; p < 0.011) with HBV infection in HD patients.

Conclusion

HCV and HBV infections are more common among HD patients in Yemen than in most Middle Eastern countries. However, the current prevalence rates are declining compared with previously published data among Yemeni HD patients. The duration of HD and number of blood units were independent risk factors for HCV infection, and patients with a history of cupping were identified as having an increased risk of HBV infection. These findings underscore the need to implement strict infection control in HD units.

## Introduction

Hepatitis C virus (HCV) and hepatitis B virus (HBV) are the most common causes of chronic disease worldwide, contributing significantly to cirrhosis and liver cancer [1] and increasing mortality [2]. According to the WHO, nearly 254 million people worldwide were infected with HBV and 50 million were infected with HCV in 2022, which represents a major public health problem [3]. HBV and HCV virus infections are more prevalent in patients on chronic maintenance hemodialysis (HD) than in the general population [4].

The incidence of chronic kidney disease (CKD) is increasing worldwide, and CKD is becoming one of the main issues in public health. In 2017, an estimated 850 million people globally lived with CKD, which is double the expected prevalence of diabetes [5]. Approximately 4 million people in the world with kidney failure or end-stage renal disease (ESRD) are treated with kidney replacement therapy (KRT), and HD remains the most common form of KRT, accounting for approximately 69% of all cases of KRT and 89% of all cases of dialysis [6].

HD patients are at an increased risk of bloodborne infections, including HBV and HCV. This could be the result of HD patients exchanging supplies and medications, as well as reusing dialyzers. In addition, there is a predisposition for nosocomial transmission during dialysis, transplantation, and the potential necessity for blood transfusions [7]. Furthermore, renal failure results in disturbed immune functions that interfere with a patient’s ability to eliminate these viruses [8].

A recent systematic review reported that the global prevalence of HCV in dialysis patients was 24.3% [9]. Notably, this figure is 14 times greater than the prevalence of HCV infection in the general population, which was estimated at 1.8% in a recent systematic review [10]. On the other hand, a recent meta-analysis of 322 studies (795,623 cases) estimated that the prevalence of HBV infection among HD patients was 7.32% [11]. Furthermore, Kenfack-Momo et al., in their recent meta-analysis, reported that the highest prevalence of HCV among HD patients was in Indonesia (63.6%), Romania (55.3%), and Egypt (55.2%) [9]. Previous systematic reviews across Middle East countries reported that the overall prevalence of HCV infection among HD patients was 25.3%, with low rates in Iraq (20%), Saudi Arabia (19%), Palestine (18%), and Lebanon (9%), and high rates in Egypt (50%), Syria (42%), and Yemen (40%) [12]. However, the prevalence rates vary across countries and are highest in underdeveloped countries [13].

Nonetheless, Yemen is a developing country, and the prevalence rates of HBV and HCV among Yemeni HD patients are underrepresented in clinical studies. Therefore, this study aimed to determine the prevalence of HBV and HCV infections among Yemeni patients on HD in the Ibb Governorate and to identify the risk factors for infections in such patients.

## Materials and methods

Study design and patients

This was a cross-sectional study of 374 Yemeni patients (223 males [59.6%] and 152 females [40.4%]) with ESRD who routinely underwent HD from January 2019 to August 2022 at the Nephrology Unit of Al-Thawra Hospital in Ibb city, Yemen. The clinical characteristics, demographic data, and risk factors associated with HBV and HCV infection, including a history of blood transfusion, a history of surgical procedures, duration of dialysis, dental extraction, Hijama, and injection, of all patients were collected through a questionnaire, and their medical records were reviewed.

Sample processing

Blood samples (5 mL) were collected from every patient under sterile conditions and centrifuged (Beckman Coulter, Inc., Brea, CA, USA), and then the sera were stored at −20°C until analysis. Later, the serum was assessed for the detection of antibodies against HCV virus (HCV-Ab) and hepatitis B surface antigen (HBsAg) via an enzyme-linked immunosorbent assay (ELISA) supplied by Biocare Diagnostics Ltd., Zhuhai, China, according to the manufacturer’s instructions, at the Medical Laboratory, University of Science and Technology (MLUST), and laboratories of Al-Thawra Hospital, Ibb, Yemen.

Statistical analysis

All the statistical analyses were performed using SPSS Version 21.0 (IBM Corp., Armonk, NY, USA). Demographic variables are presented as the mean ± standard deviation (SD), median and interquartile range (Q1, Q3) for skewed variable, or frequency and percentage, as appropriate. The chi-square test and Fisher’s exact test were performed to differentiate the proportions between subgroups as appropriate. Logistic regression analysis was conducted via the Enter method, with HBsAg and anti-HCV antibodies used as the dependent variables and all possible risk factors as independent variables. Statistical significance was defined as p < 0.05.

## Results

The study included 374 patients, and their mean age was 43±15 years. There were 223 (59.6%) males and 152 (40.4%) females. The median dialysis duration was 24 (11-69) months, and the percentage of patients on dialysis for less than 12 months was 34.2%, while 26.2% and 39.6% had a longer duration of dialysis (12-36 and >36 months, respectively). Only nine (2.4%) patients had previous renal transplantation. A total of 337 (90.1%) patients had received blood transfusions while on HD, and the transfused blood originated mainly from the General Hospital blood bank. The main presumed etiologies of ESRD were hypertension (58.8%), diabetes mellitus (14.1%), and others. Patients’ demographics and clinical characteristics are shown in Table 1.

**Table 1:** Sociodemographic and clinical characteristics of the study participants The values are presented as the mean ± SD, medians (Q1, Q3), or frequencies and percentages. ESRD, end-stage renal disease; HBV, hepatitis B virus; HCV, hepatitis C virus; HD, hemodialysis

Characteristics		Frequency	%
Age (years)	Mean ± SD	43±15	
Gender	Male	223	59.6
	Female	152	40.4
Duration of hemodialysis (months)	Median (interquartile range Q1, Q3)	24 (11, 69)	
	≤12 months	128	34.2
	13-36 months	98	26.2
	>36 months	148	39.6
Blood transfusion during HD	Yes	337	90.1
	No	37	9.9
Number of transfused blood units	Median (interquartile range Q1, Q3)	10 (3, 21)	
Kidney transplantation	Yes	9	2.4
	No	365	97.6
History of surgery	Yes	148	39.6
	No	226	60.4
History of using parenteral medications (injection)	Yes	165	41.1
	No	209	55.9
Hepatitis B vaccination	Yes	34	9.1
	No	340	90.9
History of dental treatment	Yes	109	29.1
	No	265	70.9
History of blood transfusion	Yes	71	19
	No	303	81
History of cupping therapy (Hijama)	Yes	327	87.4
	No	47	12.6
Family history of HCV	Yes	17	4.5
	No	357	95.5
Family history of HBV	Yes	1	97.7
	No	373	0.3
Dialysis at more than one center	Yes	204	54.5
	No	170	45.5
ESRD etiology	Hypertension	220	58.8
	Diabetes mellitus	53	14.1
	Other causes	78	20.9
	Unknown	23	6.1
Blood replacement	No	11	2.9
	Erythropoietin	26	7
	Blood transfusion only	138	36.9
	Erythropoietin and blood transfusion	199	53.2

Prevalence of HBV and HCV infections among hemodialysis patients

The overall prevalence of HBV infection (positive HBsAg) was 6.15% (n=23), and the prevalence of HCV infection (positive HCV antibodies) was 31% (n=116). Coinfection with HBV/HCV was detected in 0.8% (n=3) of patients (Table 2).

**Table 2:** Prevalence of HBV and HCV among hemodialysis patients The values are presented as frequencies and percentages. HBsAg, hepatitis B surface antigen; HBV, hepatitis B virus; HCV, hepatitis C virus

Viral infection		Frequency	%
HCV antibody	Positive	116	31
HBsAg	Positive	23	6.15
Co-infection (HCV antibody and HBsAg)	Positive	3	0.8
Negative		232	62
Total		374	100

Distribution of HCV and HBV infection among hemodialysis patients

Table 3 shows the distribution of HCV and HBV infections among HD patients. The results revealed that positive HCV and HBV infections were more prevalent in HD patients with long durations of dialysis (p = 0.001 and p = 0.033, respectively). Furthermore, the prevalence rates of HCV infection in our study were significantly higher in HD patients who had a history of blood transfusion (p = 0.023). On the other hand, patients who had a history of cupping therapy (Hijama) were more likely to be positive for HBV infection than other patients (p = 0.016) (Table 3).

**Table 3:** Distribution of HCV and HBV infection among hemodialysis patients The results are given as frequencies and percentages. P-values were calculated using the chi-square test and Fisher’s exact test. *Fisher exact test. HBV, hepatitis B virus; HCV, hepatitis C virus

Characteristics	Positive HCV antibody		Positive HBsAg	
	N (%)	P-value	N (%)	P-value
Gender				
Male	63 (28.3)		18 (8.1)	
Female	53 (35.1)	0.160	5 (3.3)	0.060
Duration of hemodialysis (months)				
≤12 months	18 (14.1)		4 (3.1)	
13-36 months	35 (35.7)		4 (4.1)	
≥36 months	63 (42.6)	0.001	15 (10.1)	0.033
Blood transfusion during HD				
Yes	107 (31.8)		18 (5.3)	
No	9 (24.3)	0.354	5 (13.5)	0.064*
Kidney transplantation				
Yes	3 (33.3)		1 (11.1)	
No	113 (31.0)	0.99*	22 (6.0)	0.439*
History of surgery				
Yes	49 (33.1)		11 (7.4)	
No	67 (29.6)	0.92	12 (5.3)	0.403
History of blood transfusion				
Yes	30 (33.1)		5 (7.0)	
No	86 (29.6)	0.92	18 (5.9)	0.728*
History of using parenteral medications (injection)				
Yes	51 (243)		11 (6.7)	
No	65 (28.4)	0.023	12 (5.7)	0.712
History of dental treatment				
Yes	35 (32.1)		8 (7.3)	
No	81 (30.6)	0.769	15 (5.7)	0.539
Hepatitis B vaccination				
Yes	6 (17.6)		0 (0)	
No	110 (32.4)	0.077	23 (6.8)	0.249*
History of cupping therapy (Hijama)				
Yes	17 (36.2)		7 (14.9)	
No	99 (30.3)	0.414	16 (4.9)	0.016

Associations of HCV and HBV infection with possible risk factors among hemodialysis patients

The associations of HCV and HBV infection with independent risk factors were evaluated among HD patients. Logistic regression analysis revealed a significant association between an increased number of units of blood transfused (OR = 3.1; 95% CI: 1.7-5.6; p < 0.001), a long duration of dialysis (OR = 3.2; 95% CI: 1.9-5.2; p = 0.001), and HCV infection in HD patients. On the other hand, a history of cupping therapy (Hijama) was a significant risk factor associated with OR = 3.4 (95% CI: 1.3-8.77; p < 0.011) and with HBV infection in HD patients (Table 4).

**Table 4:** Associations of HCV and HBV infection with possible risk factors among hemodialysis patients Data are presented as ORs (95% CIs). Logistic regression analysis was used to estimate the associations of HCV and HBV infection (dependent variables) with possible risk factors (independent variables). *Log-transformation for continuous variables HBV, hepatitis B virus; HCV, hepatitis C virus

Characteristics	Positive HCV antibody		Positive HBsAg	
	OR (95% CI)	P-value	OR (95% CI)	P-value
Gender				
Male	Ref		Ref	
Female	0.73 (0.47-1.34)	0.161	0.39 (0.14-1.07)	0.069
Age years	1.0 (0.99-1.02)	0.351	1.1 (0.98-1.01)	0.729
Duration of hemodialysis (months)				
≤12 months	Ref		Ref	
≥12 months	4.1 (2.3-7.1)	<0.001	2.59 (0.86-7.79)	0.089
Duration of hemodialysis (months)*	3.2 (1.9-5.2)	<0.001	1.1 (0.37-3.3)	0.857
Number of transfused blood units*	3.1 (1.7-5.6)	<0.001	1.1 (0.37-3.3)	0.857
Blood transfusion				
No	Ref		Ref	
Yes	1.5 (0.66-3.2)	0.356	0.361 (0.13-1.03)	0.059
Kidney transplantation				
No	Ref		Ref	
Yes	1.13 (0.27-4.5)	0.879	1.95 (0.23-16.3)	0.358
History of surgery				
No	Ref		Ref	
Yes	1.13 (0.27-4.5)	0.479	1.43 (0.62-3.33)	0.405
History of using parenteral medications (injection)				
No	Ref		Ref	
Yes	1.01 (0.65-1.6)	0.964	1.38 (0.59-3.2)	0.455
History of dental treatment				
No	Ref		Ref	
Yes	1.07 (0.67-1.74)	0.769	1.32 (0.54-3.21)	0.54
History of cupping therapy (Hijama)				
No	Ref		Ref	
Yes	1.30 (0.69-2.5)	0.415	3.4 (1.3-8.77)	0.011
Dialysis at more than one center				
No	Ref		Ref	
Yes	1.48 (0.95-2.32)	0.084	1.3 (0.56-3.12)	0.531

## Discussion

HCV and HBV infections in patients undergoing HD continue to be major global health concerns, even though advanced HD delivery has improved over the past decade [6]. Yemen is a developing country that has one of the highest prevalence rates of HCV among HD patients. According to a meta-analysis performed by Kenfack-Momo et al., the prevalence rate was reported to be 40% after that reported in Egypt and Syria, which had the highest reported rates across Middle Eastern countries [12]. This study estimated the prevalence of HCV and HBV infection in the Ibb Governorate, Yemen.

Our study demonstrated that the overall prevalence of HCV was 31%, whereas that of HBV was 6.15%. Among HD patients in the Ibb Governorate, 0.8% had coinfections with HCV and HBV. Notably, the current HCV prevalence is lower than that reported in a recent meta-analysis performed by Kenfack-Momo et al. in 2024, which reported a prevalence of more than 40% [9], and that reported in previous studies conducted in the Aden governorate, which reported a prevalence of 62.7% in 2010 [14] and 40.2% in 2015 [15]. Notably, the current HBV prevalence rate is notably lower than that reported in previous studies carried out in different governorates among Yemeni dialysis patients, which reported that the prevalence rates of HCV and HBV were 56.3% and 43.6%, respectively, in Taiz [16] and 46.01% and 48.83%, respectively, in Zabeed [17]. Several factors may be the causes of this decline in the prevalence of HBV and HCV among Yemeni HD patients, such as increased adherence to infection control procedures, improved HIV, HBV, and HCV screening tests for both blood donors and dialysis patients via ELISA technique, and reduction in the need for blood transfusions because of increasing erythropoiesis-stimulating agents use. On the other hand, our results were higher than the rates reported by a previous study conducted between 2013 and 2016 among 100 patients undergoing HD in Ibb city, Yemen, by Almezgagi et al., who reported that the prevalence of HBV was 3% and that of HCV was 21% [18]. These low rates may be due to the small sample size.

However, the prevalence rates of HCV and HBV in our study were higher than those reported from neighboring Arab countries, such as Saudi Arabia (1.67% and 3.77%, respectively) [19], Syria (3.2% and 22.1%, respectively) [20], Palestine (7.4% and 3.8%, respectively) [21], Somalia (3.2% and 7.3%, respectively) [22], and Sudan (8.5% and 4.5%, respectively) [23]. Conversely, our study revealed a lower HCV prevalence than that reported in other developing countries, such as Indonesia (63.6%), Egypt (55.2%) [9], Kosovo (53%) [24], and Pakistan (38.75%) [25]. This heterogeneity based on nationality may result from the absence of a framework for minimum and optimal safety and quality standards for HD as well as different approaches and gaps in kidney failure care between countries [26].

The present study also revealed the long duration of dialysis associated with HCV and HBV infection in HD patients. These findings are similar to those of previous reports [20,22,23,27,28]. In addition, a number of units of blood transfused were associated with HCV infection in HD patients, which is in agreement with other studies [22,23] but is not associated with HBV infection in our study; this finding is consistent with the findings of [20,23], in contrast with the study by Jeele et al. [22]. On the other hand, a history of cupping therapy (Hijama) was a significant risk factor associated with HBV infection in HD patients. This finding is in accordance with that of Rehman et al. [29], who reported that the practice of cupping (Hijama) transmission is a risk factor for HBV.

We acknowledge the limitations of our study. Genotypes of HCV and HBV were not evaluated, and despite the relatively large sample size of the study, a detailed analysis of relationships with current HBV infections was hampered by the small number of HBV-positive samples.

## Conclusions

In summary, our study revealed that HCV and HBV infections are more common among HD patients in Yemen than in most Middle Eastern countries. However, the current prevalence rate is declining compared with previously published data among Yemeni HD patients. The duration of HD and number of blood units were associated with HCV infection, whereas a history of cupping was associated with HBV infection. The results underscore the need to implement strict programs for HCV and HBV prevention and testing to curtail transmission in HD units.
